# Nanosafety Analysis of Graphene-Based Polyester Resin Composites on a Life Cycle Perspective

**DOI:** 10.3390/nano12122036

**Published:** 2022-06-14

**Authors:** Francisco Aznar Mollá, Jose Antonio Heredia Alvaro, Oscar Andreu Sánchez, Carlos Fito-López, Inmaculada Colmenar González

**Affiliations:** 1 Research Line Sustainable Chemistry and Supramolecular Chemistry, University of Jaume I Castellón, 12071 Castellón de la Plana, Spain; al363139@uji.es; 2 Industry Chair Director 4.0., University of Jaume I Castellón, 12071 Castellón de la Plana, Spain; heredia@uji.es; 3 Laboratory of Ecotoxicology and Environmental Quality, Department of Cellular Biology, Functional Biology and Physical Anthropology, Faculty of Biological Sciences, University of Valencia, Dr. Moliner 50, 46100 Valencia, Spain; 4 ITENE, Technological Institute of Packaging, Transport and Logistics, 46001 Valencia, Spain; inmacolmenar@yahoo.es

**Keywords:** nanotechnology, nanomaterial, nanoparticle, ecotoxicity, risk assessment, graphene

## Abstract

The use, production, and disposal of engineering nanomaterials (ENMs), including graphene-related materials (GRMs), raise concerns and questions about possible adverse effects on human health and the environment, considering the lack of harmonized toxicological data on ENMs and the ability of these materials to be released into the air, soil, or water during common industrial processes and/or accidental events. Within this context, the potential release of graphene particles, their agglomerates, and aggregates (NOAA) as a result of sanding of a battery of graphene-based polyester resin composite samples intended to be used in a building was examined. The analyzed samples were exposed to different weathering conditions to evaluate the influence of the weathering process on the morphology and size distribution of the particles released. Sanding studies were conducted in a tailored designed sanding bench connected to time and size resolving measurement devices. Particle size distributions and particle number concentration were assessed using an optical particle counter (OPC) and a condensation particle counter (CPC), respectively, during the sanding operation. A scanning electron microscope/energy dispersive X-ray (SEM/EDX) analysis was performed to adequately characterize the morphology, size, and chemical composition of the released particles. A toxicity screening study of pristine and graphene-based nanocomposites released using the aquatic macroinvertebrate Daphnia magna and relevant human cell lines was conducted to support risk assessment and decision making. The results show a significant release of nanoscale materials during machining operations, including differences attributed to the % of graphene and weathering conditions. The cell line tests demonstrated a higher effect in the human colon carcinoma cell line Caco2 than in the human fibroblasts (A549 cell line), which means that composites released to the environment could have an impact on human health and biota.

## 1. Introduction

Expectations in the use of engineered nanomaterials (ENMs) have exponentially grown over the last decade. Their unique physicochemical properties enable the development of new products with extraordinary properties, which include size, shape, or surface area, and which can lead to adverse health effects [1].

Graphene and its derivatives are currently being explore for a multitude of applications in electronics, photocatalysis, sensors, medicine, plastics, or construction [2,3,4]. In addition, new applications of graphene and its derivates such as biomedicals applications, or its use in a wearable respiration sensor among others, have been recently published [5,6]. Considering the large commercial interest in graphene products and their fast expansion, safe and sustainable development of graphene-related materials (GRMs) in technologies and products requires close attention due to the potential impact of these materials on human health and the environment [3].

The release of graphene particles during the manufacture, use, and end-of-life may reach the environment, including the atmosphere, and aquatic or terrestrial compartments, where the variety of target species and physical chemical factors creates a complex challenge. In fact, some studies have shown evidence of the potential effects of graphene-related materials on aquatic and terrestrial organisms [7,8]. In general, there are still large knowledge gaps with respect to graphene’s environmental and toxicological effects [9,10]; the (eco)toxicological information than can be extracted from the few developed studies is not adequate to be applicable to real case scenarios in which consumers use graphene-related polymer composites as components of final products.

In this sense, the evaluation of their potential release has gained importance in recent years, motivating the need to better understand the potential hazards posed by the production and use of polymer-based nanocomposites. A list of activities associated with a high potential for releasing particles has been identified in the literature including diffusion, desorption, or matrix degradation. The latter is related to processes such as mechanical abrasion, thermal degradation, hydrolysis, and UV exposure [11]. The release of particles associated with these activities might be particles of a matrix alone, free-standing particles, partially protruding or fully embedded particles in the polymer matrix, or in limited cases, in the form of dissolved ionic compounds. Since the form and nature of the particles released may lead to different human/environmental effects, several studies have focused on identifying the nature and extent of nanoparticles and nanoparticle-containing fragments released from nano-enabled products because of weathering [12,13]. Evaluations conducted to date do not indicate a high propensity for discreet nanomaterial release, but rather composite particles of a matrix with partially or fully embedded nanomaterial [14]. The few available studies have reported a release of particles with an aerodynamic diameter smaller than 10 µm, which can penetrate to the alveolar region of the lung [15]. Particles with a size of ~200 nm have also been identified in sanding operations [16].

As mentioned above, the evaluation of the activities with a potential release of nanomaterials is important in order to establish control and safety measures to prevent exposure to nanomaterial particles of workers, consumers, and any person who may been in contact with these materials during their life cycle. Generally, the steps in the life cycle of nano-enabled products are divide as follows: (1) material extraction, (2) synthesis of the ENM, (3) nanocomposite production, (4) product manufacturing, (5) use, (6) end of life, and (7) recycling [17].

**Figure 1:**
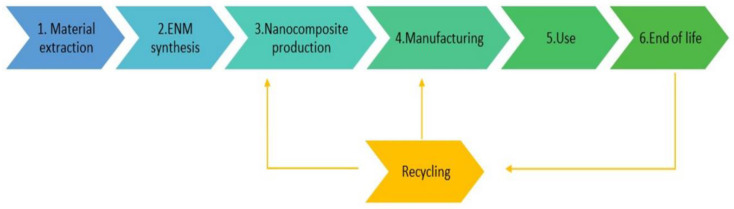
Life-cycle steps of nano-enabled products.

These steps are presented in the scheme shown on Figure 1. As mentioned above, some data are available on graphene exposures [4,18,19,20]. These studies have mainly focused on the synthesis stage in pilot plants [20,21], leading to a lack of information on exposure to particulate matter throughout its life cycle.

The aim of this study is to present a set of measurements for the release of particles in Stages 5 and 6 of the life cycle. For this porpoise, two different scenarios were studied: (1) abrasion and weathering of a pioneering graphene-based polyester resin and (2) use of painting spray containing graphene material. 

Moreover, the characterization of aerosol particles released after mechanical abrasion on graphene-reinforced epoxy composites under different UV weathering conditions was also conducted.

Once composites are produced and marketed, they are exposed to several machining processes such as cutting, drilling, and sanding. Nanomaterial release from nanocomposites during these types of processes has received special attention in the literature when it takes place at the manufacturing stage, since it they are a potential source of particle release. In addition, high volumes of nanocomposites are handled in these processes [16,17]. However, there is a lack of information regarding the release of particles during machining processes in the use of the final product (Step 5, Figure 1).

Additionally, one of the current challenges in the paint and coating industry is to apply nanotechnology to achieve environmentally friendly and durable coatings.

One of the most promising anti-corrosion technologies is the addition of graphene nanoplatelets (GNPs) into paints and coatings. This modification potentially reduces the usage and application time for paint and the effort required to extend the life of capital assets such as bridges or automotive vehicles. In this sense, applied graphene materials (AGM) and related materials should to be considered in the occupational and environmental impact of using paints, coatings, and propellant cans containing graphene [22].

As mentioned above, the evaluation of activities with potential release of nanomaterials is important in order to establish control and safety measures to prevent exposure from nanomaterial particles of workers, consumers, and any person who may been in contact with these material during their life cycle. The aim of this study is to present our first safety assessment of a pioneering graphene-based polyester resin with usages on a wide range of applications, highlighting the building sector. Specifically, the assessment focuses on: (1) the characterization of aerosol particles released after mechanical abrasion on graphene-reinforced epoxy composites, as well as the analysis of the exposure potential during the application of graphene based coatings; (2) the quantification of the amounts of protruding and free-standing graphene platelets in the abraded particles, and (3) the assessment of the potential effects of the pristine and abraded graphene particles released on the aquatic micro invertebrate Daphnia magna and human cell lines. Daphnia magma was selected due to its importance as a model organism in various biological disciplines, from aquatic ecology to biomedical sciences. The cell lines A549 and Caco-2 were selected due to the relevance for improving current knowledge on the cellular response to foreign particles deposited in the lungs or in the gastrointestinal tract due to an accidental uptake by biota, which signify acute inhalation and oral uptake toxicity in vitro.

## 2. Materials and Methods

### 2.1. Materials

Graphene powder and related polyester resin were provided by Graphenglass (www.graphenglass.com, accessed on 15 April 2022). The material consisted of graphene particles of 10–55 µm size formed by 2–6 graphene sheets with a BET surface > 250 m^2^/g. The properties of graphene are listed in Table 1.

The BET surface area was determined by nitrogen physisorption at 196 °C (model AS1, Quantachrome, Boynton Beach, FL, USA). The sample in the form of dried powder was first degassed for 24 h at 200 °C.

### 2.2. Synthesis of the Graphene-Polyester Composite

The manufacturing process of the nanocomposite followed four phases: silanization of graphene oxide, resin dispersion, curation, and post-curation.

The silanization of the grafene oxide was done in a water solution via magnetic agitation and sonic treatment. In this case, 1 g of MPS (methacryl oxypropyl-trimethoxysilane) was dispersed dropwise in 125 mL of distilled water and 1–3 drops of ClH were added. The aqueous solution was agitated for 15 min at a controlled temperature. Next, 125 g of grafene oxide was added progressively, while maintaining the magnetic agitation for 30 min. The solution of grafene was agitated in a magnetic mixing device. Optimization and control of silane addition, processing times, sonic power, and exact temperature control are essential in the silanization process. In this case, the combination was realized through a 10 h process of sonication (with 20 kHz frequency), maintaining the mixture in vibration, and following alternate 20 min cycles of operation and shutdown at 65 °C. The resulting functionalized grafene oxide was dried at 110 °C for 4 h to remove water, solvents, and reaction by-products.

The dispersion of the resin was performed with the addition of initiators and catalyzers. Following the curing cycle, a post curing cycle was needed to consolidate the resulting vitrification in the resin increasing the temperature after gelation. To prepare the composite at different wt%, polyester resin was dispersed into functionalized grafene oxide to obtain samples with the different percentages of the silanizated grafene relative to the polymeric resin. The resin was dispersed progressively for 20 min under magnetic vibration. To initiate the polymerization reactions, methyl ethyl ketone and 1–8 wt% of cobalt octoate 6% was added (depending on the grafene proportion), and stirred for 2 min at 40 °C. The combination was sonicated for 30 min (with 20 kHz frequency) maintaining the mixture in vibration, followed by alternate 2 min cycles of operation and shutdown, maintaining a controlled temperature.

After curing for 24 h at 20 °C, the composite was post cured for 4 h at 60 °C in order to increase the polymer vitrification and to obtain the resulting nanocomposite.

Table 2 depicts the list of graphene-polyester composite samples produced and analyzed under the scope of the present study.

### 2.3. Physicochemical Characterization of Graphene-Based Resins

The rheological properties of the polyester resin and the resulting composite were determined with a rheometer (TA instruments, AR2000, Barcelona, Spain) operated in cone and plate arrangements (stainless steel cone, with a 40 mm diameter and a 4° cone angle) at 25 °C.

Morphology and thickness of the composite pieces were determined by cross-section and surface SEM using a Hitachi 4800 microscope Toledo, OH, USA. The FTIR spectra were evaluated on a Perkin Elmer 781 spectrophotometer, Madrid, Spain, on the powder samples between 400 and 4000 cm^−1^. Typical properties of the resin were as follow: specific gravity measured at 25 °C of 1.10, viscosity at 25 °C (Rhéomat 37.35 s^−1^) from 2.0 to 2.5 dPas, acid value from 19 to 23 mg KOH/g, and volatile content from 41 to 45. Table 3 depicts the main physicochemical properties of the curated graphene-based resins.

### 2.4. Weathering Process

For the weathering process, a UV chamber was used to simulate the environmental degradation conditions. The test was carried out in an accelerated weathering instrument purchased from the Heraeus Company (Model Suntest CPS+, Hanau, Germany). A two-phase approach was conducted, including a first phase to replicate dry atmospheric conditions at 50 °C, followed by an immersion process, where the temperature of the black body was automatically assigned as a function of the irradiance, to simulate wet atmospheric conditions. The testing program applied included alternative cycles of wet and dry conditions of 500 h for 20 days. The same process was repeated to reach a total weathering time of 1000 h. Table 4 summarizes the weathering process conditions. 

### 2.5. Abrasion Process and Particle Collection

In this study, a sanding beach including and a mechanical sander and tailored designed clamping system to guarantee a homogeneous sanding process to promote reproducibility was applied. Temperature (range 20–25 °C) and humidity (range 40–50% relative humidity) were monitored. A bosch sander (Model Bosch PBS 75 AE, Madrid, Spain) equipped with a sanding belt was used to simulate the sanding process on the surface of the graphene-based composites. A medium (G/K 120) grit-sized sanding paper from the brand Piranha (Black & Decker, Madrid Spain) was applied to the sanding machine.

**Figure 2:**
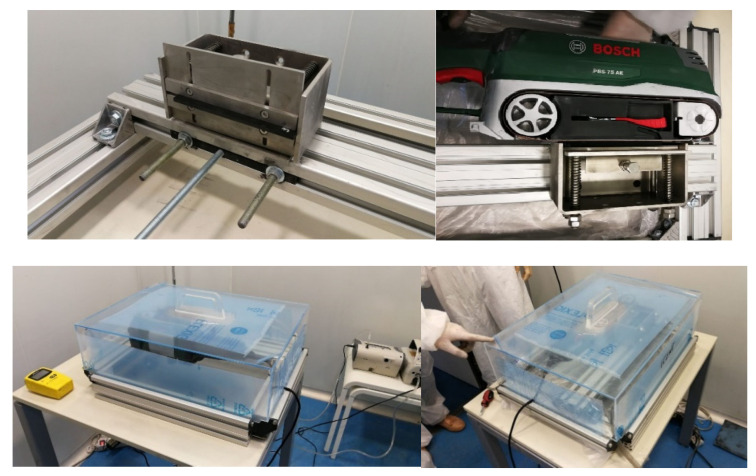
Sanding bench for abrasion and particle collection.

The graphene-based composite pieces were sanded at the low-speed position of the sander for 20 s. The released particles from the abrasion/sanding area were collected in a stainless aluminum tray, which had been placed under the sanding belt. Released particles were analyzed using a multimetric measurement approach. Two instruments were used, including an optical particle sizer (OPS) (Model 3330, TSI, Shoreview, MN, USA) and a condensation particle counter (CPC) (Model 3077, TSI, Shoreview, MN, USA). In parallel, the particles were collected on polycarbonate filters with a pore size of 0.2 μm mounted on 37 mm open-faced cassettes. Figure 2 shows photographs of the sanding bench.

The morphology of the pristine and the released particles from the composites was characterized using a HITACHI S-1800 Toledo, OH, USA scanning electron microscope (SEM) equipped with EDX Toledo, OH, USA to analyze the elemental composition of the materials released.

The background concentration, generated by the running abrasion module without sample contact but with the sanding machine operating (Figure 1), was investigated before the studies to characterize the level and nature of the background particle concentrations.

The inlets of the devices were located at a height of 10 ± 0.2 cm and ~0.5 m from the source of the particles (sanding belt) using flexible Tygon^®^ (Tecnyfluor, Barcelona, Spain) tubes attached to the inlets of the instruments. The main specifications of the suite of instruments used are listed in Table 5. The background concentration in the room was measured without sanding and with the sander turned off.

### 2.6. Spray of Paints Containing Graphene Nanoparticles (GNP)

Five cans of paint (~2700 g each) of different viscosities (high, medium, and low), which were free of or containing GNPs types of paints, were tested. The paints were mixed before spraying with a curing agent.

Each paint sample was mixed with the curing agent within its own can and mixed with a pint mixer connected to a drill. After few minutes of mixing, the can was feed immediately to an airless spray machine (Standard Hi-Boy Series—Graco, Minneapolis, MN, USA) at constant pressure (3000 psi) with 3 different tip sizes (415, 421, zand 427) depending on the viscosity of the paint (see Table 6) and sprayed at around 50 cm from a surface placed at the end of the exposure chamber.

Sampling filters, 37 mm in size and made of PVC and PTFE, were used to deposit particles collected in the air and to characterize chemical composition, aggregation state, and shape by microscopy techniques (SEM/EDX).

Between tests, up to 7 liters of cleaning solvents with toluene, methyl acetate, and xylene was flushed through the spray gun to clean the conduits. The fact that the last paint did not contain graphene and was sprayed after paints with graphene served to test if graphene particles from previous processes could still bew found after cleaning and spraying.

The background was also tested before the spray process to compare with the particles present in the ambient before the tests.

### 2.7. Ecotoxicity 

For the ecotoxicity studies, the Daphtoxkit F™ bioassay (Microbiotest, Ghent, Belgium) was used to estimate the effect of pristine and abraded graphene particles on the crustacean *D. magna*. Daphnia are planktonic crustaceans, characteristic of fresh water, with a size range between 0.5 and 3 mm. The acute toxicity tests were performed according to the Toxkit^®^ protocol. The Daphtoxkit™ F test was performed in accordance with test procedures of the OECD Guideline 202 (OECD, 2004) and ISO 6341 (UNE—EN ISO 6341, 2012).

The test was performed on neonates born from Epiphyas (resistance eggs) of Daphnia 72–80 h at a controlled temperature of 20–22 °C and light (6000 lux) in the OECD 202 medium previously aerated. The neonates placed in the trial, therefore, did not exceed 24 h of life. The Daphnia were fed spirulina during the two hours prior to the assay. The assay was carried out in conditions of darkness and at a temperature of 23 °C.

To calculate the median lethal concentration (LC50), 24- and 48-hour EC50 values, as well as their associated 95% confidence intervals (95% CI) were calculated using the EPA-probit v1.5 program (USEPA).

A standard reference toxicity test with K_2_Cr_2_O_7_ was run in parallel to each test series to verify the sensitivity of the *D. magna*.

### 2.8. Toxicity Studies

**(a) Respiratory impact** Several assays were applied to the in vitro lung models to measure the levels of cytotoxicity (cell death, or a reduction in cell viability), oxidative stress, and inflammation. 

The A549 cell line was selected as a representative cell of the alveolar epithelium of the gas-exchange region of the lung. The A549 epithelial cells were grown in continuous culture in DMEM medium containing 10% FCS (Life Technologies, Alcobedas, Madrid, Spain), non-essential amino acids (diluted from 100× stock solution, Sigma, St. Louis, MO, USA), sodium pyruvate (1 mM, Life Technologies, Alcobedas, Madrid, Spain), L-glutamine (diluted from 100× stock solution, Life Technologies, Alcobedas, Madrid, Spain), penicillin (100 U, Sigma, St. Louis, MO, USA), and streptomycin (100 μg/mL, Sigma, St. Louis, MO, USA). This was designated complete medium. Cells were removed from culture by trypsinization and plated into 96-well plates at 2 × 10^5^ cells/mL (100 μL/well). The plates were incubated for 24 h at 370 °C, the wells were washed with medium, and treatments were added to each well in a final volume of 100 μL in complete medium.

**(b) Oral Route—ingestion** As in the previous case, several assays were applied to the in vitro Caco-2 cell model to measure the levels of cytotoxicity (cell death, or a reduction in cell viability), oxidative stress, and inflammation that occurred in response to exposure to pristine and abraded materials.

Caco-2 cell lines were cultivated for 10 days and then seeded at densities of 105 to 106 cells per ml (0.1 mL per well) in a 96-well plate by trypsinizing and centrifuging at 8.4× *g* for 5 min. After a 24-h exposure, cell viability was measured using the conventional 3-(4,5-dimethylthiazol-2-yl)-2,5-diphenyltetrazolium bromide (MTT; ATCC) reduction assay.

In both, fibroblasts and Caco-2, the ability of each material to generate reactive oxygen species (ROS) was determined using the DCFH-DA assay. Dichlorodihydrofluorescin diacetate (DCFH-DA) is able to cross cell membranes and enter cells where it is cleaved to form DCFH. The presence of ROS causes oxidation of DCFH into DCF which is fluorescent and can be evaluated to indicate a change in oxidative stress. ROS values are expressed as the percentage of fluorescence intensity relative to the control wells.

**Figure 3:**
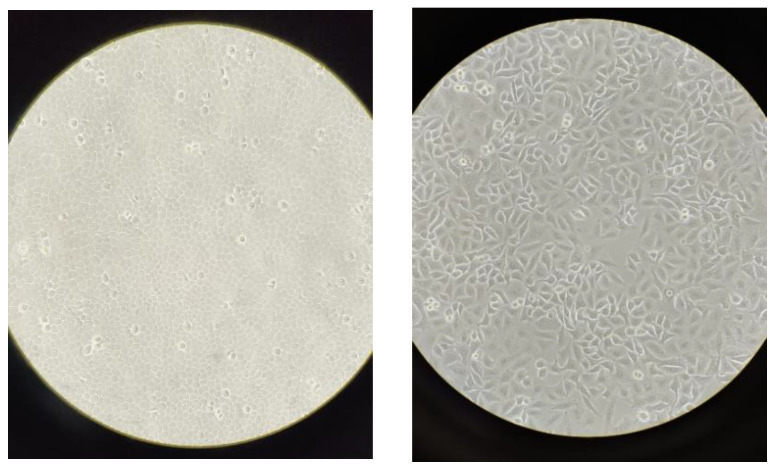
(**Right**) A549 cell line micrograph; (**Left**) Caco-2 cell line.

Figure 3 shows a micrography of the cell lines used, taken using an inverted microscope.

## 3. Results and Discussion

### 3.1. Abrasion Test

**Figure 4:**
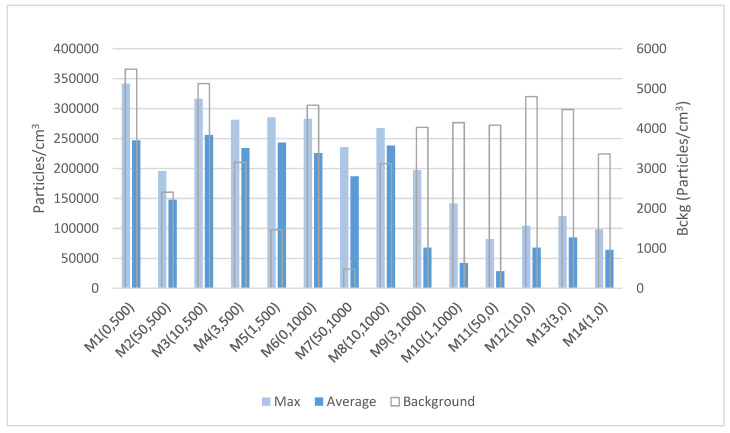
Concentration of particles measured with a CPC during the sanding activity. Maximum and average concentration values are referred to along the *Y* axis.

Figure 4 shows the variations in the particle number concentration for the 14 samples processed during the operation. The minimum concentration reflects the background levels of particles before starting the sanding operation, reaching values below 6 × 10^3^ particles cm^−3^, which can be derived from the grey column depicted in it (secondary axis).

A large increase immediately after the beginning of the sanding operation was observed; the highest obtained peak values reached up to 3.5 × 10^5^ particles cm^−3^, which was about 50 times higher than the background levels.

The concentration of particles in the nano range measured by the CPC showed that weathered samples released a higher amount of submicron particles (Ø < 1 µm), especially significant for samples subjected to a 500 h aging process, where the maximum peaks were observed. Such an issue denotes an alteration of the polymer surface, allowing a higher rate of particle release.

The differences between aging processes (1000 h vs. 500 h) were not significant, therefore, it has been suggested that the polymer’s surface layer plays a relevant role in the exposure potential. 

The levels of particles released for no-weathered samples was relatively low, with particles reaching a peak of ~1.4 × 10^5^ particles cm^−3^ (for sample M10), and an average particle level concentration of 6 × 10^5^ particles cm^−3^. This was in contrast to the high average levels found for weathered samples, with average levels of 2.2 × 10^5^ particles cm^−3^ and 1.35 × 10^5^ particles cm^−3^ for samples subjected to a weathering period of 500 and 1000 h, respectively.

**Figure 5:**
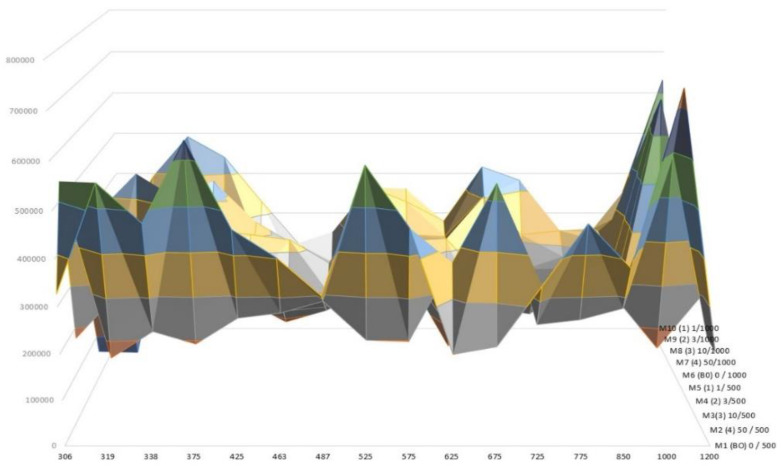
Size distribution of particles measured during the sanding process.

**Figure 6:**
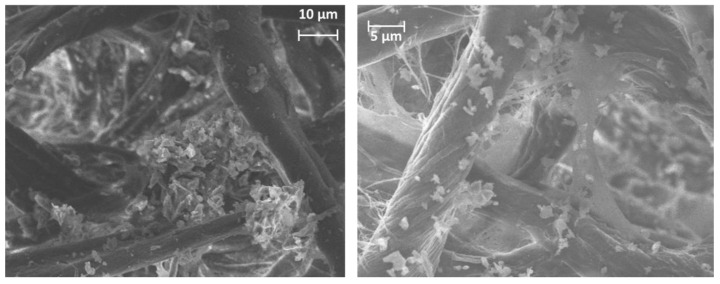
Detail of SEM images including particles collected in filters during the abrasion process.

The analysis of the data measured by the particle sizer (TSI OPS 3300) showed different modes corresponding with particles with an average particle size of ~320 ± 2 nm, ~540 ± 2 nm, and ~1150 ± 2 nm. The maximum peaks were observed for particles above 1 µm (Figure 5), being mainly due to the number of particles embedded in the polymeric matrix, as can be derived from the SEM picture depicted in Figure 6.

The SEM images show only wear particles from the resin material. Therefore, it can be assumed that the abraded resin is the main source of the particles released. There was no free-graphene particles collected. These graphene nanoparticles were embedded in the matrix or attached to the surface of these particles indicating the importance of measuring micrometric-sized particles in addition to nanosized particles. These observations highlight the fact that the use of a matrix when handling nanomaterials greatly minimizes the exposure of workers to airborne nanoparticles.

The SEM/EDX analysis of sander emission also revealed a reasonable amount of carbon agglomerates ranging from 0.1 to 20 μm.

A large number of particles collected in the filters. Figure 6 includes an EDX spectrum identifying the elements that are represented in the filtration media. Carbon, oxygen, and aluminium were the elements identified with an abundance of ~80%, 18%, and 1%, respectively.

**Figure 7:**
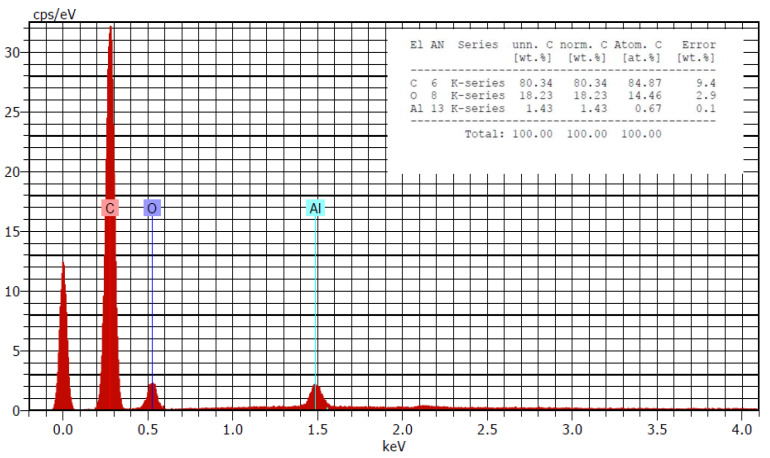
SEM/EDX analysis of particles collected in filters during the abrasion process. Elements identifed: carbon (C), oxygen (O) and aluminium (Al).

Figure 7 includes an EDX spectrum identifying the elements that are represented in the filtration media.

### 3.2. Paint Spraying

Real-time devices were used to characterize the size of the particles and droplets emitted during spraying. Three different devices were used, placed at fixed positions near the target surface (open face), except for one device which was portable and was fitted to the operator within their particle breathing zone (PBZ). The real-time particle devices included two TSI 3007 CPCs, an OPS TSI 3330, and an Aerotrak (TSI 9350, Shoreview, Minnesota, USA) counter. Mass concentrations and mass-based size distributions were estimated by calculations from particle number concentrations and number-based size distribution data.

As mentioned above, PVC and PTFE filters were used to collect the material deposited during spray tests and being analyzed by SEM-EDX.

**Figure 8:**
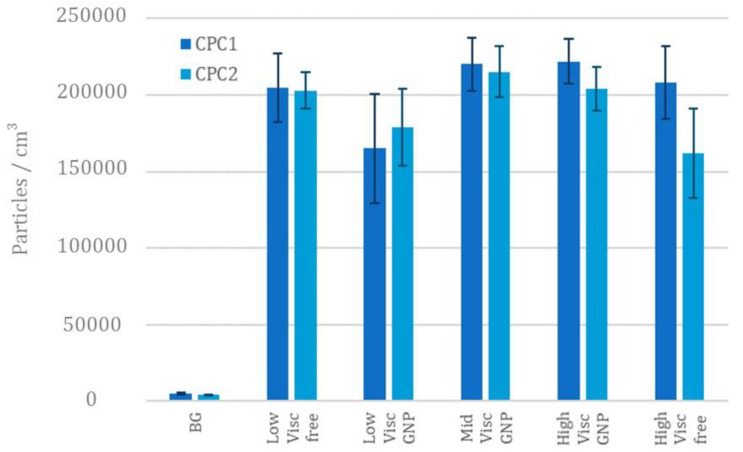
Comparison of the mean PNC of both CPCs.

Figure 8 shows the particle number concentration (for sizes <1 µm) obtained with two CPCs when low, mid, and high viscosity graphene containing paints were sprayed in the testing room. In addition, the free-graphene paints were also tested in order to compare the difference in the particle distribution when graphene was formula present in the paints.

Although the number of particles released during spraying was 2 to 3 orders of magnitude greater than that of the background, the number of particles under the micron fluctuated around 2 × 10^5^ particles/cm^3^. In addition, we observed that there was no remarkable difference in the concentration due to the viscosity of the paints. The particle size distributions also had similar trends regardless of whether the paint contained graphene or not. Based on this data, it was not possible to discriminate the paints that did or did not contain the GNP. The difference between the measures of the two CPCs is within the deviation of the measures, thus, they are comparable and the spray within the exposure chamber is uniform.

**Figure 9:**
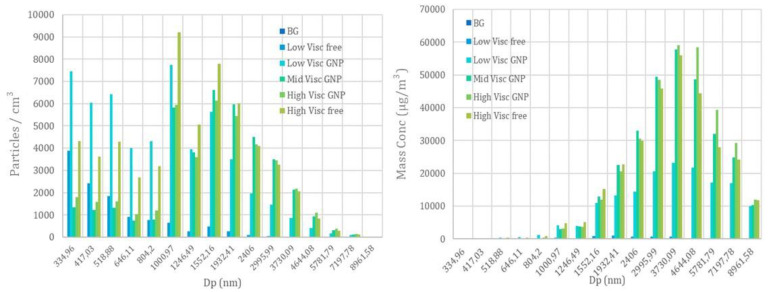
Plot of the particle number concentration (**left**) and mass concentration taken from the OPS device (**right**).

The results obtained from the OPS device showed a similar behaviour for the mid and high-viscosity paints containing GNPs, while the low viscosity showed a higher release of particles below the micron and a lower release of particles above the micron. Although the low viscosity free paint has a distribution shifted to particles below 1 µm, the other paints are up to three orders of magnitude greater than the background, and a multimodal distribution appears with peaks at 0.5, 1.0, 1.7 and 3.0 µm (Figure 9).

A similar behaviour was found for the Aerotrak results, where the low viscosity, free-GNP paint has a distribution shifted to the smaller diameters with the highest concentrations, while in the other cases, particles between 5 and 25 µm do not exceed the 1000 particles.

Regarding the mass distribution obtained with the OPS device, there are clear peaks at 1.0, 1.8–2.0, 3.7, and 7.1 µm, which coincide with the previous peaks (recall that submicron particles have no mass and large particles occur in much lower concentrations).

**Figure 10:**
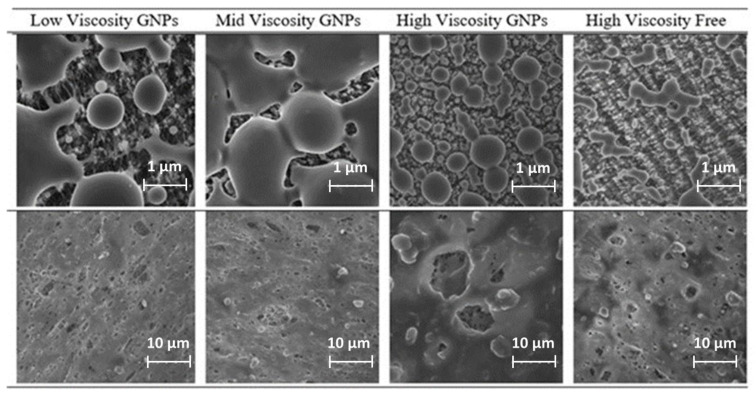
SEM images for the PTFE (**upper row**) and PVC (**lower row**) filters collected.

For the analysis of the impact filters by SEM, no free GNPs were observed for airless spraying. The GNP particles observed were always coated by a layer of paint and the higher the viscosity of the paint the thicker the coating of the GNP. Borders appear to be rounder on higher densities, although further testing should be made to determine the composition and thickness of this coating. Figure 10 shows the SEM images for the two types of filters collected.

Due to the smoother surface of the PVC filter, particles are more easily deposited in them, while in the PTFE, mainly drops remain, despite having a smaller pore size. The gathered particles in PVC filters are integrated onto a layer of paint in most cases.

From the background analysis, a contribution of ambient particles is observed. This contribution consists of particles with different shapes and compositions, probably dust, composed mainly of different metals. In the elemental analysis, silicon and iodine appear, in addition to the elements of the filter and the constant presence of aluminum through all samples. However, the EDX analysis seems to not be enough to determine the composition of the elements present, due to the difficulty of discerning the background and filter elements from the carbon of the particles. 

Particle sizes were calculated by Image J and appear along with the images with a number indicating the length of each particle in microns. It can be seen that the particles detected in the PVC filters have diameters between 7 and 15 µm.

### 3.3. Ecotoxicity Analysis

The results on the aquatic toxicity of graphene showed that the immobility and mortality of the microcrustacean Daphnia magna was not affected, indicating a low acute toxicity in micro invertebrates. These results indicate that no alterations were identified at the organism level, however, alterations at the cellular level such as increased ROS generation cannot discarded. It should be noted that the lack of altered individual responses may suggest low ecological consequences related to exposure to graphene composites.

Table 7 depicts the effect concentration levels (EC) for the samples analyzed.

### 3.4. Toxicity Analysis 

**Figure 11:**
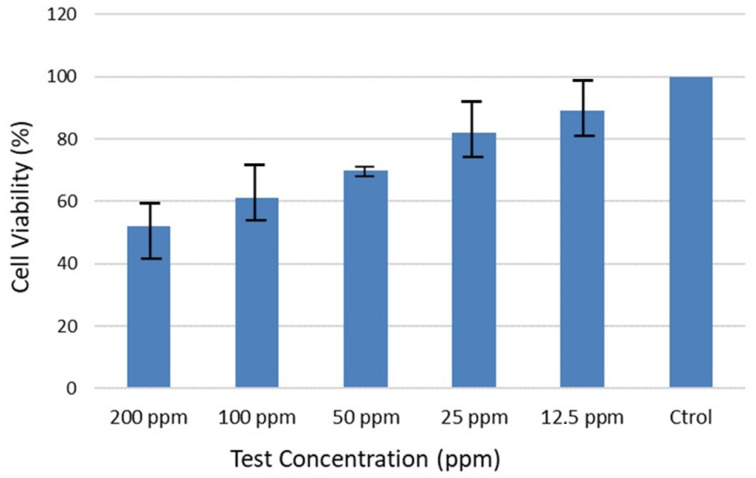
Effect of graphene on the generation of cytotoxic effects in human skin fibroblast cells.

The cell viability showed a dose-dependent pattern for pristine graphene to A549 cells in the concentration range of 1.5 to 200 ppm, with a first effect at a concentration higher than 25 ppm (cytotoxicity above 80%), which indicates a low toxicity potential of graphene in this cell-free system. This dose-dependent effect of the particles can be found in Figure 11, where the effect of the particles over time at different doses is depicted. For example, for 50 ppm of graphene, the cell viability was 71% after 24 h of exposure.

**Figure 12:**
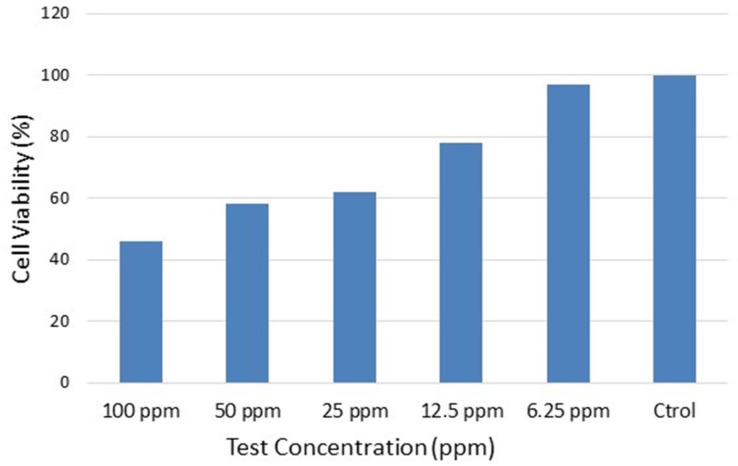
Effect of graphene on the generation of cytotoxic effects in the colon adenocarcinoma cell line.

Concerning the Caco-2 cell line, the cell viability also showed a dose-dependent pattern for pristine graphene in the concentration range from 6.25 to 100 ppm, with a first effect at a concentration higher than 12.5 ppm (cytotoxicity above 60%), which indicates a low to moderate toxicity potential of graphene in this cell-free system. This dose-dependent effect of the particles can be found in Figure 12, where the effect of the particles over time at different doses is depicted. For example, for 100 ppm of graphene, the cell viability calculated was 46.33% after 24 h of exposure. This means that the cytotoxic potential for the oral route is higher than the one observed for the A549 cell line.

Table 8 shows the effect concentration values calculated, including lower and upper limits. The dose range chosen is expansive in order to include relevant exposure concentrations (lower range) but also higher concentrations to allow EC50 values to be calculated for comparison and ranking purposes. On the basis of the data retrieved using A549 and Caco-2 cells, the toxicity for graphene was estimated to be 205 and 68 ppm for the inhalation and oral route, respectively, which means that the oral route is of prime importance. 

**Figure 13:**
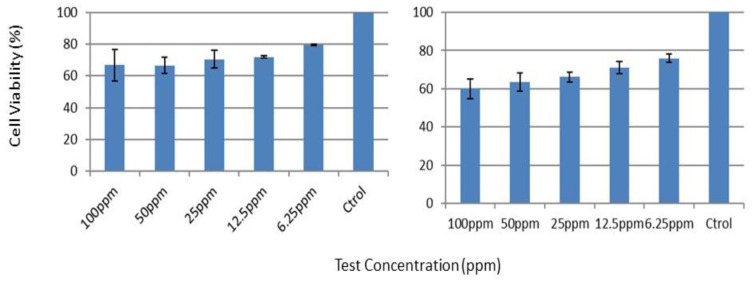
Effect of graphene-related composites on the generation of cytotoxic effects in human skin fibroblast cells: (**Right**) Pristine nanocomposite; (**Left**) graphene-based composite sample (M14, 50% of graphene).

Figure 13 shows the effects of the graphene-related composites on the A549 cells. Our results suggest that cell viability was not decreased by the pristine composite and the graphene-based composite sample, which was in agreement with other recent reports that showed non-toxicological effects of graphene-related composites. The EC50 values were above 100 ppm, which means that the samples tested could be considered to be non-toxic. These results also suggest that the graphene-based composite induced much less response in the A549 cells than that of the graphene nanosheets.

**Figure 14:**
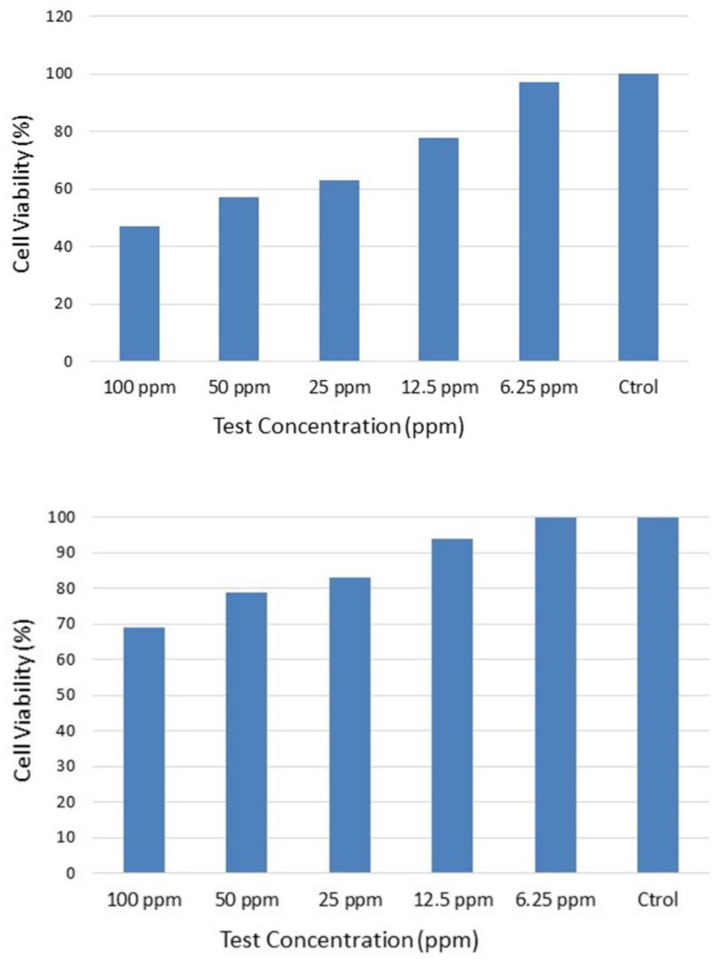
Effect of graphene-related composites on the generation of cytotoxic effects in Caco-2 cells: (**Top**) Pristine nanocomposite; (**Bottom**) graphene-based composite sample (M14, 50% of graphene).

Similarly, Figure 14 shows the effects of the graphene-related composites on the Caco-2 cells. A similar behavior to the one observed for the A549 cells can be identified. Notwithstanding, a higher level of decrease was observed for the Caco-2 cell line. The EC50 values were above 100 ppm, which means that the samples tested could be considered to be non-toxic. These results also suggest that the graphene-based composite induced much less response in the A549 cells than that of the graphene nanosheets.

**Figure 15:**
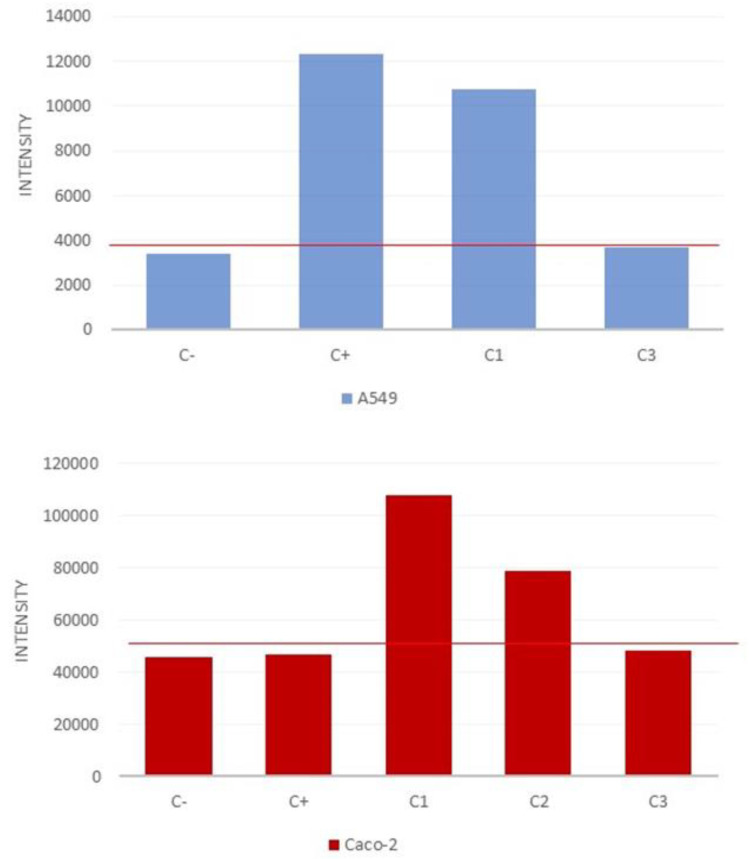
ROS generation in the cell lines A549 (**top**) and Caco-2 (**bottom**)after an exposure period of 24 h. Concentrations: C1, 20 ppm; C2, 10 ppm; C3, 5 ppm.

Regarding the ROS generation, both cell lines show a base production, identified in Figure 15 as negative control C-. In the case of the A549 cell line, a concentration of up to 20 ppm in the media generated a significant response in the cell, however, the tested concentrations of 10 and 5 ppm did not cause an increase above the C- response. The Caco-2 cell line was more sensitive to graphene, with a high increase in the ROS generation at 10 ppm, where the A549 did not show any adverse response.

The results on the toxicity of graphene and graphene composite samples are summarized in Table 9.

## 4. Conclusions

In the present work, we aimed to assess the current level of human and environmental hazard of GBMs and to emphasize the importance of understanding the structure and the activity relationships that underlie the potential toxicity of these materials.

A safe assessment for products based on graphene derivatives is essential because of the huge number of applications of these products in aircraft, wind turbines, bridges, ships, cars, and sports equipment, to name a few.

In addition, the toxicological and release potential of graphene and graphene-based resins was investigated considering inhalation as the main route of exposure for risk assessment purposes.

Furthermore, real-time measurements demonstrated a high release of particles below 1 µm; however, these particles, according to the SEM images, were rather made up from matrix materials, which contained the embedded particles. It was clearly visible that a higher concentration level was obtained for weathered samples, which suggested a significant impact of the weathering process on the release of nano- and micro-sized particles. The particle size distribution did not show differences in the size of the particles released, suggesting that the size of the particles could be related to the specifications of the sanding device applied.

Our data suggest that graphene-related composites can be considered to be safe materials considering the inhalation route.

The release of particles of different sizes related to the spraying of the paints was observed, showing an increase in particle concentration and mass with respect to background concentrations. In addition, the contribution of ambient particles of different shapes and compositions, probably dust and materials from the spraying process composed mainly of different metals, was also observed.

No remarkable difference in the particle concentration due to the viscosity of the paints was found, moreover, no population of free-graphene nanoparticles was identified by SEM microscopy in the filters sampled from any of the tested paints. In all cases, particles seem to be coated by a thick layer of paint.

Nevertheless, the EDX analysis seems to be not enough to determine the composition of the elements present. Further testing would be necessary to analyze the composition and thickness of the layers.

The toxicity studies also concluded that graphene and graphene resins have a low toxicity profile. However, more investigation is needed to specify sublethal effects, including ROS and inflammation. Nevertheless, it is hoped that as this framework is populated with additional studies, ideally using GMRs that have undergone rigorous characterization, including the structure–activity relationship of these materials. Indeed, it is also important to move from a descriptive to a predictive toxicological model in order to be able to use these promising GRMs in multiple applications in a safe context for humans and the environment.

## Figures and Tables

**Table 1:** Properties of the graphene incorporated into the polymeric matrix.

Property	Description/Value
Flake thickness (nm)	0.8–2 (2–6 monolayers)
Particle size (µm)	10–55
Brunauer−Emmett−Teller (BET) surface area (m^2^/g)	>250
Purity	>98%
Specific gravity (20 °C)	1.18

**Table 2:** List of graphene-polyester composite samples.

Sample ID	Description	Picture (Nanocomposite Pieces)
M1 (B0) &0	Graphene%: 0 Weathering (h): 500	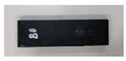
M6 (B0) &0	Graphene%: 0 Weathering (h): 1000	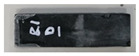
M11 (4) &0	Graphene%: 0 Weathering (h): 0	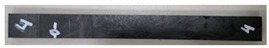
M5 (1) JH91	Graphene%: 1 Weathering (h): 500	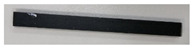
M10 (1) JH91	Graphene%: 1 Weathering (h): 1000	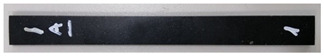
M14 (1) JH91	Graphene%: 1 Weathering (h): 0	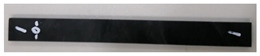
M4 (2) JH92	Graphene%: 3 Weathering (h): 500	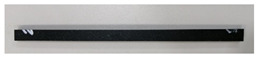
M9 (2) JH91	Graphene%: 3 Weathering (h): 1000	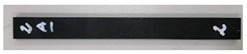
M13 (2) JH91	Graphene%: 3 Weathering (h): 0	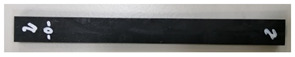
M3 (3) JH93	Graphene%: 10 Weathering (h): 500	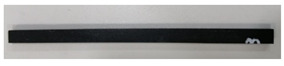
M8 (3) JH91	Graphene%: 10 Weathering (h): 1000	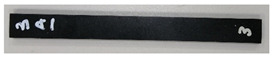
M12 (3) JH91	Graphene%: 10 Weathering (h): 0	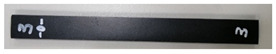
M2 (4) JH94	Graphene%: 50 Weathering (h): 500	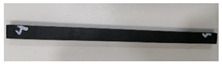
M7 (4) JH94	Graphene%: 50 Weathering (h): 1000	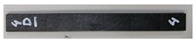

**Table 3:** Properties curated graphene-based resins.

Property	Value (Curated Composite)
Young’s modulus	3800 MPa
Fracture strain	2%
Tensile strength	60 Pa
Hardness (GYZJ 934-1)	40
Volatile content	41–45%
Water absorption (24 h/23 °C)	15
Heat deflection temperature	70 °C
Specific gravity (20 °C)	1.18 2 C
Acid value	19–23 mg KOH/g

**Table 4:** Weathering process conditions in Test 1 and Test 2. Minimum (Min), Average (Av) and Maximum (Max) values are listed.

Test	Hours	Temperature (°C) (Min/Av/Max)	HR (%) (Min/Av/Max)
1	481 h	24.6/62.62/82.9	5.5/10.21/43.4
2	537 h	18.5/58.23/84.9	7.0/18.81/72.3
Total	1018 h		

**Table 5:** Instrumentation used in the measurement campaign.

Sample ID	Function	Specifications
Condensation particle Counter Model 3007 (CPC) (TSI)	Quantify the particle number concentration	Size range, 0.01 to > 1.0 µm
Optical particle sizer (OPS) Model 3330 (TSI)	Classifies in concentration of number and particle size	Concentrations up to 10^5^ particles/cm^3^
Apex air sampling Pump (Casella pump)	Obtain samples in filtering media to elucidate chemical nature by SEM	Size range, 0.3–10 μm in up to 16 channels

**Table 6:** Spraying characteristics for each paint.

	Low Visc Free	Low Visc GNP	Mid Visc GNP	High Visc GNP	High Visc Free
**Pressure (psi)**	3000	3000	3000	3000	3000
**Tip size**	415	415	421	427	427

**Table 7:** Ecotoxicity measured as EC50 values at 24 and 48 h.

	% GR/Trat.	EC_50_ mg/L—24 h	EC_50_ mg/L—48 h
N0206	Pure/pristine	318	146
M1 (B0)	0%/500 h	>100 (516)	>100 (304)
M2 (4)	50%/500 h	>100 (358)	>100 (221)
M6 (B0)	0%/1000 h	>100 (437)	>100 (251)
M7 (4)	50%/1000 h	>100 (298)	>100 (157)
M11 (4)	1%/0 h	>100 (381)	>100 (240)
M14 (1)	50%/0 h	>100 (216)	>100 (142)

**Table 8:** Toxicity measured as EC50 values at 24 and 48 h.

	Value	Low Limit	Upper Limit
EC50 A549	205 ppm	140.7	376.5
EC50 Caco2	68 ppm	36.1	1245.0

**Table 9:** Toxicity results for samples analyzed.

	% GR/Trat.		A549	Caco-2	
		EC_50_ mg/L—24 h	ROS	EC50 mg/L—24 h	ROS
N0206	Pure/pristine	205	+C1	68	+C2
M14 (1)	50%/0 h	68	+C1	>100	+C2

## Data Availability

Not applicable.
